# FAST—framework for AI-based surgical transformation

**DOI:** 10.3389/fdata.2025.1655260

**Published:** 2025-09-12

**Authors:** Harmehr Sekhon, Farid Al Zoubi, Paul E. Beaulé, Pascal Fallavollita

**Affiliations:** ^1^Division of Geriatric Medicine, Department of Medicine, St. Mary's Research Centre, McGill University, Montreal, QC, Canada; ^2^Centre for Addictions and Mental Health (CAMH), Ontario, Canada and Gerontology Institute, University of Massachusetts Boston, Boston, MA, United States; ^3^School of Electrical Engineering and Computer Science, University of Ottawa, Ottawa, ON, Canada; ^4^Division of Orthopaedic Surgery, The Ottawa Hospital, Ottawa, ON, Canada; ^5^Interdisciplinary School of Health Sciences, University of Ottawa, Ottawa, ON, Canada

**Keywords:** operating room, prescriptive analytics, artificial intelligence, surgical data science, clinical translation

## Abstract

**Background:**

The use of machine learning (ML) in surgery till date has largely focused on predication of surgical variables, which has not been found to significantly improve operating room efficiencies and surgical success rates (SSR). Due to the long surgery wait times, limited health care resources and an increased population need, innovative ML models are needed. Thus, the Framework for AI-based Surgical Transformation (FAST) was created to make real time recommendations to improve OR efficiency.

**Methods:**

The FAST model was developed and evaluated using a dataset of n=4796 orthopedic cases that utilizes surgery and team specific variables (e.g. specific team composition, OR turnover time, procedure duration), along with regular positive deviance seminars with the stakeholders for adherence and uptake. FAST was created using six ML algorithms, including decision trees and neural networks. The FAST was implemented in orthopedic surgeries at a hospital in Canada's capital (Ottawa).

**Results:**

FAST was found to be feasible and implementable in the hospital orthopedic OR, with good team engagement due to the PD seminars. FAST led to a SSR of 93% over 23 weeks (57 arthroplasty surgery days) compared to 39% at baseline. Key variables impacting SSR included starting the first surgery on time, turnover time, and team composition.

**Conclusions:**

FAST is a novel ML framework that can provide real time feedback for improving OR efficiency and SSR. Stakeholder integration is key in its success in uptake and adherence. This unique framework can be implemented in different hospitals and for diverse surgeries, offering a novel and innovative application of ML for improving OR efficiency without additional resources.

## 1 Introduction

The use of machine learning (ML) to optimize surgical efficiency is an opportunity to bridge the gap between care required and current capacity without adding additional resources. Surgery waitlist times are currently higher than pre-pandemic statistics, especially for orthopedic surgery (Canadian Institute for Health Information, 2024a,b). With the surgery needs predicted to increase as the population ages, the current 43% productivity level of the healthcare system must be improved (Fairley et al., 2019). This need is especially prominent for surgeries such as knee replacement, which has already seen an increase of 22.5% for knee replacement and 20.1% for hip replacement procedures, between 2014 and 2019 (Cram et al., 2018). With stretched healthcare budgets and the addition of more resources not being possible for most, optimizing operating room (OR) time and space which alone has been reported to inflate surgical costs by 30%, is needed (Fairley et al., 2019).

The use of ML models for surgeries is not new. Current approaches to improving surgical efficiencies in hospitals, and reducing wait times includes using ML to analyse hospital data, increased government funding, increasing number of joint operating room (OR) days, and overlapping swing rooms (Beaulé et al., 2015; Waly et al., 2020). However, the OR output or Surgical Success Rate (SSR), which is the rate of surgeries completed on time remained low (39%) with current approaches, with close to 60% of surgery days having overtime (Gold et al., 2023). Current non-clinical ML algorithms focus on data to predict a certain aspect of an operation; this can be predicting an event before surgery, after surgery, or even the duration of the surgery itself (Maheshwari et al., 2017; Schiele et al., 2021; Bartek et al., 2019). Of the three categories of predictions, procedure duration is the most common. However, these approaches do not capture the multifaceted nature of OR efficiency in terms of actual performance or provide real-time recommendations tailored to the team and surgery department, rendering them incomplete (Jain et al., 2020; Sculley et al., 2015). There is a need for an individualized and iterative ML framework that can improve OR efficiency by providing real-time recommendations, using existing data, resources and personnel. It is also essential to assess the feasibility of integrating such a framework in a clinical setting, to assess the clinical teams' acceptance and adherence to the ML recommendations.

The Framework for AI-based Surgical Transformation (FAST) is designed to go beyond mere metric forecasting, by directly impacting OR throughput, such as surgery duration. The FAST model is unique in that it not only captures OR target predictions, but also specific recommendations that can lead to them. This framework is equipped with decision support systems for the non-clinical settings and is designed to be flexible and adaptable, allowing for repurposing across various surgical procedures and healthcare environments. The overall aim of FAST is to assist diverse healthcare institutions and professionals in identifying the most suitable optimization framework for their needs. These institutions rely on ML models with varying data input streams, creating a versatile set that caters to a wide range of healthcare settings. This flexibility allows institutions to utilize the framework leveraging the data they currently possess even if a healthcare institution has access to only one aspect of data, such as patient metrics or specific steps in surgical procedures.

## 2 Overview of FAST

FAST was designed to improve Operating Room output with the use of data and a Prescriptive Analytics System (PAS), which serves as a decision support system.

**Figure 1:**
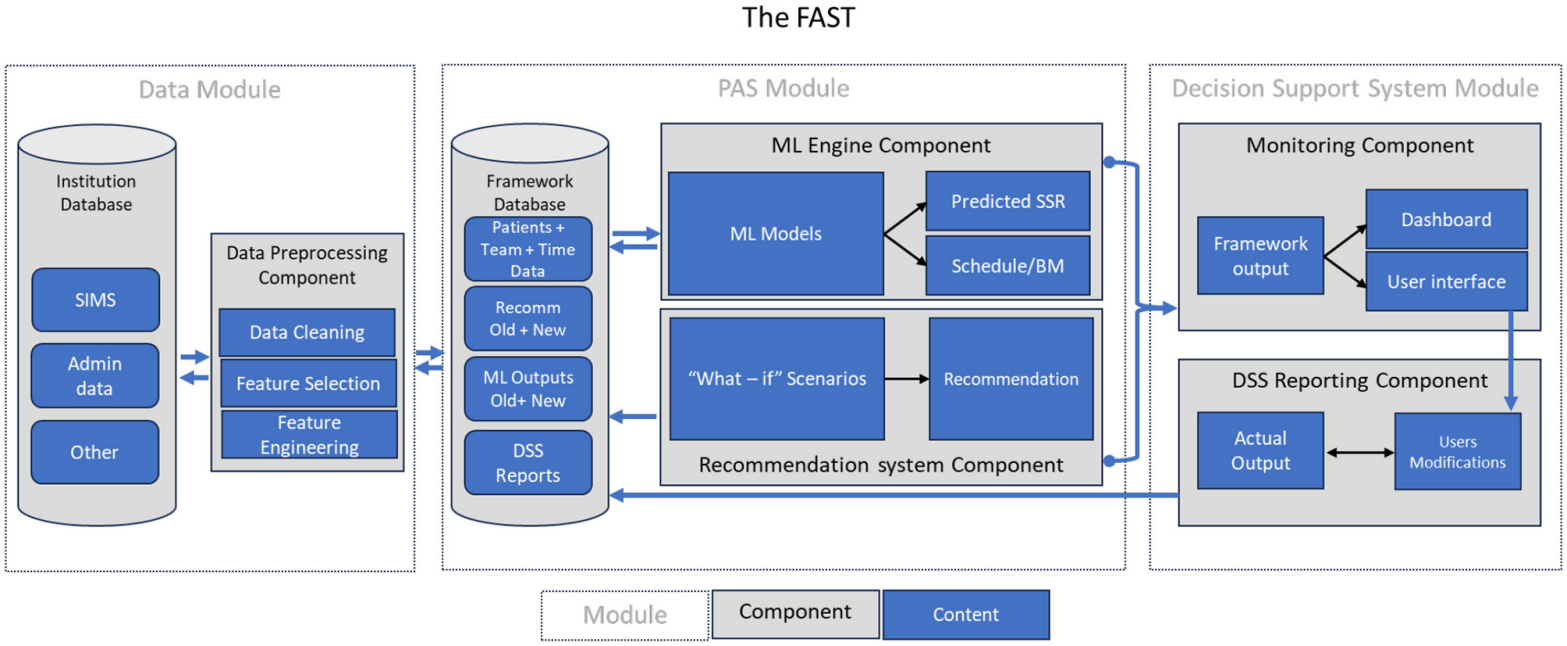
FAST components and building blocks.

This section provides a comprehensive elucidation of each component of FAST, including the input, output, building modules, and the necessary elements for each component to constitute a fully functional FAST. Figure 1 illustrates the fundamental modules involved in constructing the FAST (Al Zoubi, 2024). The process initiates with the Data Module, followed by the PAS Module and ending by the Decision Support System (DSS) module.

### 2.1 The data module

The Data Module (DM) are comprised of two primary components, the institution's database and data preprocessing component. The institution's database [e.g. Surgical Information Management Systems (SIMS)] contains the necessary data (e.g. patient information, time metrics) for the framework module. The data preprocessing component extracts the essential data for each the framework module, cleans it, and performs feature engineering when necessary. The output of the data module is refined, ready-to-use, and selected metrics for the framework module. The role of the framework database is to manage and store the processed and categorized data provided by the DM.

### 2.2 The PAS module

Once the DM completes the data preparation, it is subsequently sent to the PAS Module (PM), which consists of three components: the ML engine, the framework database, and the recommendation system.

The ML engine component houses all the ML models necessary for the PM. The output of these models includes the predicted OR throughput and the specific values for the categories (e.g., surgery time, patient info) required to attain specific OR throughput.

The recommendation system component function is to implement a recommendation which contributes to transforming the framework into a prescriptive analytics decision support system.

### 2.3 Decision support system module (DSSM)

The creation of FAST into a fully developed product, requires a well-functioning framework with a tested and implemented recommendation system, and additional components. These include a user interface, a dashboard, and database integration for both the input/output data of the machine learning model and the output of the system after each run.

The user interface serves to streamline the clinical teams' interaction with the FAST. As users utilize the dashboard to monitor the progression of surgery over time, they gain the capability to intervene in real-time. This involves allowing administrators to manually modify inputs, adjust scheduling, modify benchmarks, and either accept or reject suggestions from the system. The framework database also serves as a repository for ML output data, what-if scenarios, selected recommendations, and the actual data received at the completion of each stage by the Decision Support System Module (DSSM).

## 3 FAST implementation

To assess FAST in a real-world clinical environment it was deployed with orthopedic surgeries, focusing on arthroplasty procedures in Ottawa, Canada, REB was not required as this was a quality improvement project under the Ottawa University REB. This version of FAST was named as Workflow Monitoring FAST (WM-FAST).

### 3.1 Overview of arthroplasty surgeries: from pre-op to post-op

This is a short overview to provide a better understanding about the Canadian surgery scheduling and pre/post operation (OP).

Patients are referred from family physicians, and undergo evaluations from physiotherapists and surgeons prior to scheduling surgeries. Surgery waitlists vary from 6-weeks to 8-months, and are organized based on time of consenting as well as priority level based on degree/severity of pain and disability.

The surgeon is allocated several surgery days per month to perform surgeries within the public hospital which ultimately dictates the wait time for the patient's surgery. The surgery booking is done 6–8 weeks before. There is an anesthesia assessment 2 weeks ahead of the surgery as well as an education session with a physiotherapist to prepare for post-op which is often the same day as the surgery. The patient arrives to the in the surgical day care unit (SDCU) 2 h before the surgery. They receive pre-operative pain medications as well as antibiotics and then anesthesia in the OR suite. After the surgery is completed, the patient is brought to the post anesthesia care unit (PACU) to be monitored for 1–2 h, and then transferred back to surgical day care unit to be mobilized with physiotherapy and ensure they can transfer independently. Further instructions are provided for self-monitoring for complications as well as when to follow-up with their surgeon.

In respect to clinical staff allocation, circulator “nurses” in the SDCU, OR suites and PACU are trained and specialized for their areas of work. In other words, a nurse working in SDCU is not necessarily trained/qualify to work in PACU. Nurses in the OR suite require specialized training that take several months. The anesthesiologists are randomly assigned to the OR suite 1–2 days ahead of the surgery and are not necessarily the same anesthesiologist that assessed the patient in the PACU.

The four categories of data input to the AI-driven framework include (1) time metrics (anesthesia preparation time (APT), Anesthesia (start, ready, stop and finish) time, time in room, surgical preparation time, case start/finish, surgery procedure and finish time, turnover, time out of the room, case number, date); (2) Staff (team) metrics (Surgeon, anesthesiologist, circulator nurse); (3) Patient Metrics (campus, type of surgery and anesthesia, sex, age, BMI, ASA); and (4) Safety metrics (90-day readmission, reason for readmission, length of stay).

### 3.2 WM-FAST: data module

The goal of the WM-FAST is to improve efficiency and achieve a higher OR throughput by monitoring and optimizing the time taken by each step of a well-defined healthcare process/procedure. The data for the WM-FAST is collected from four joint ORs which is defined as a scheduled 8-h day (7:30 AM−3:30 PM) where four unilateral joint replacements are performed by the same surgeon (Al Zoubi et al., 2023a). A surgical day having delays and moving into overtime (i.e. past 3:30 PM) negatively affects the SSR. Shown in Table 1 are the patient-specific surgery demographics and statistics, including indicators such as the American Society of Anesthesiologists (ASA) Physical Status Classification System (American Society of Anesthesiologists, 2020). We used the same data as in that was collected from the SIMS at our institution, please refer to this manuscript for more details about the methodology and data collection, as well as descriptive analysis (Al Zoubi et al., 2023a,b).

**Table 1:** Patient-specific surgery demographics and statistics.

**Personnel and procedure**	**Sample size**	**Patient metrices**	**Count**
Surgeons	5	Females	2,461
Circulating nurses	44	Males	2,335
Anesthesiologists	152	Average age	64.1
Four joint days	1,199	Age Range	17–99
Cases	4,796	Average BMI	29.93
Total hip arthroplasties (THA)	1,461	BMI range	17.1–51.4
Total knee arthroplasties (TKA)	1,496	Average ASA^*^	2.45
Hip resurfacing (HR)	652	ASA range	1–4
Unicompartmental knee arthroplasties (UKA)	242		
Other procedures (combination)	945		

^*^ASA: Is the American society of anesthesiologists physical status classification system, which provides an overview of the patient clinically, from 1–6, where 1 is the most medically fit.

The arthroplasty procedures we compiled data on had the following workflow steps, for more information please see our previous publication (Al Zoubi et al., 2023a):

1. Surgical preparation time (SPT)
2. Anesthesia ready
3. Surgical procedure duration (Procedure start time stamp to Procedure finish time stamp)
4. Anesthesia finish time (AFT)
5. Procedure finish to patient out of room time (OR specific time variables like cleanup, communication, post-surgery reflections, etc.)
6. Overall turnover time (It's governed by both internal and external factors, like delays in preparing the next patient for surgery that takes place outside the OR).

These six time-specific numerical inputs are logged by a circulating nurse in the SIMS of the hospital, which pre-emptively removes part of the bias associated with obtaining these variables (i.e., objective observer). These inputs are also what makes this framework procedure agnostic, as virtually every surgical procedure can be divided into multiple time-bound steps to identify the outliers.

The responsibility of generating the required time metrics as input for the ML models lies with the Data Preprocessing component in the Data module. The calculation occurs each time there is an updated value for any of the timestamps. The system's default values are set as our baseline values, corresponding to the 77% SSR benchmarks explained in (Al Zoubi et al., 2023a).

### 3.3 WM-FAST: PAS Module

#### 3.3.1 ML engine component

The ML engine component houses all the ML models necessary for each framework. We chose six machine learning algorithms to compare their performance. The algorithms are: logistic Regression (LR), (Sculley et al., 2015) Support Vector Machine (SVM), (Al Zoubi et al., 2023a) Random Forest (RF), (Al Zoubi et al., 2023b) Deep Neural Network-Artificial Neural Networks with multiple hidden layer (DNN-ANN), (Belle and Papantonis, 2021) Extreme Gradient Boosting (XGBoost): gradient-Boosted Decision Tree (GBDT), (Al Zoubi et al., 2023c) Decision Tree (DT) (Al Zoubi et al., 2024). The rationale behind choosing different models (i.e., parametric, non-parametric, ensembling, Interpretable, deep learning) was to conduct a comprehensive ML analysis from various perspectives to identify the best model outputs.

To tune hyperparameters, we employed the grid search option. The parameters of the estimator underwent optimization through cross-validated grid search across a parameter grid.

For models' evaluation, we chose six metrics to cover different ML performance evaluation aspects. AUC-ROC: is our measure of a model's performance from a classification perspective, and it evaluates performance for all possible classification thresholds. Cross Validation (CV): multiple cross-validation iterations (6 folds) were completed on the data subsets to generate valid CV accuracy numbers, and it serves as the primary measure of the overall performance of a model. Sensitivity and specificity, for per-class performance. Sensitivity or recall is how well (how frequently) a model recognizes true positives “successful days” out of total instances. While specificity is a ML model's ability to identify a true negative “unsuccessful day” and is often used in conjunction with sensitivity to evaluate how accurate a model is. Precision shows the classifier's performance for class imbalanced data. Precision allows us to evaluate the quality of a ML model's positive predictions, successful days. From an OR perspective, this means that when a model predicts a day to be successful, precision is assessing how frequently the model's predictions align with the actual successful days. Overfitting: the difference between training and testing accuracies was measured as an indicator of the future error when the model undergoes a new dataset. If the model is overfitted, it may have trouble generalizing and adapting to the new data, which may result in inaccurate classifications and predictions, presented in Tables 2, 3.

**Table 2:** Selected values for machine learning models.

**Model**	**Parameters**			
DT	Criterion	splitter	max_depth	min_samples_split
	entropy	best	16	40
RF	n_estimators	max_depth	min_samples_split	criterion
	100	40	5	gini
SVM	C	kernel	gamma	degree
	1	rbf	1	3
LR	max_iter	C	solver	verbose
	100	5	liblinear	1
DNN-ANN	hidden_layer_sizes	Activation	solver	
	10	tanh	adam	
XGBoost	loss	n_estimators	criterion	
	log_loss	50	friedman_mse	

**Table 3:** Machine learning models comparison and evaluation.

**Time monitoring (numerical)**						
**Model**	**CV Accuracy**	**AUC-ROC**	**Sensitivity (Recall)**	**Specificity**	**Precision**	**Overfitting**
LR	76%	74%	74%	84%	62%	8%
SVM	75%	81%	74%	77%	27%	6%
RF	72%	72%	66%	59%	80%	1%
DNN-ANN	70%	74%	73%	59%	64%	4%
XGBoost	70%	76%	72%	61%	60%	4%
DT	68%	67%	74%	55%	53%	5%
Standard Deviation	0.03	0.04	0.03	0.12	0.17	0.02

Limited number of supervised machine learning models offer explainability (Belle and Papantonis, 2021). The decision tree model was opted for, to illuminate the outputs of the models. It is crucial to emphasize that the decision tree (DT) may not necessarily be the best-performing model for each framework. This choice of the DT model is solely for illustration purposes, and to enhance comprehension of the inter-relationships among various elements.

The outputs generated by the ML engine component comprises predictions regarding the likelihood of a day being successful (i.e., completing four surgeries before 3:30 pm) along with the benchmarks necessary to attain specific SSR goals during a surgical day. Table 2 presented below demonstrates that when the turnover process between two consecutive surgeries exceeds 21.5 min, it can lead to a significant decrease in the SSR, from 69 to 59%, as in scenarios 3 and 4. However, this is not the situation when a comparable delay of 11 min occurs within the surgery itself, during the surgical procedure, scenarios 1 and 2. In simple terms, a 1-min delay in one stage of the surgery can have a vastly different impact on the SSR than in another stage. This complexity makes it challenging for a human to assess the significance of a minute at a specific point in the surgery without relying on the ML engine. As an additional motivation of implementing this framework, our previous work (Al Zoubi et al., 2023c) described the method of utilizing time saved from similar surgeries to accommodate additional procedures in a day, thereby reducing the arthroplasty waitlist (Al Zoubi et al., 2023d).

The output from the ML engine is expected to flow in a cascading manner, with the output of one stage influencing the subsequent one. The machine learning model must continually adapt the SSR during the course of the operational time, operating under the assumption that forthcoming stages have predefined baseline values (Al Zoubi et al., 2023a). This is why monitoring is required, as the surgeon or the OR administrator is expected to closely observe the time and react accordingly.

The day is ideally scheduled to initiate its first surgery, “case 1,” at 7:30 am with a turnover time of 0 min. If the day commences at a different time, for instance, 7:45 am, the preprocessing module calculates the turnover value as 15 minutes (7:45−7:30). Equation 1 below is employed to estimate and calculate the remaining time metrics of the case. *Metric*_*new*_ represents the estimated time for the new metric. The term “delays or gains” signifies the difference between the actual metrics value once the stage is completed and the baseline value of that metric. If the actual value is less than the baseline value, it is considered a gain, and its sign in the formula becomes a negative value. Conversely, if the actual metric value exceeds the baseline value, it is regarded as a delay.


(1) $$Metric_{new}\;=\;Metric_{baseline}+\frac{{\left(Delays\;or\;gains\right)}_{last\;stage\;}\;}{Number\;of\;Metrics_{New}}$$

For the given example, with an actual turnover value of 15 min and a baseline value of 21.5 min, we experience a gain in time of 6.5 min (“−6.5”). The subsequent metrics will then have their estimated time as their baseline values minus 6.5/4, which is 1.625 min, as outlined in diagram B-2. The assumption here is that delays and/or gains in time from the previous stage are evenly distributed among the upcoming stage.

The actual metric values are calculated at the conclusion of each stage once the required timestamp to calculate the stage is available. The ML model's input is then updated with the actual metrics values for completed stages and with the estimated values for metrics where actual values are not yet available. A SSR is generated at each new model run.

**Figure 2:**
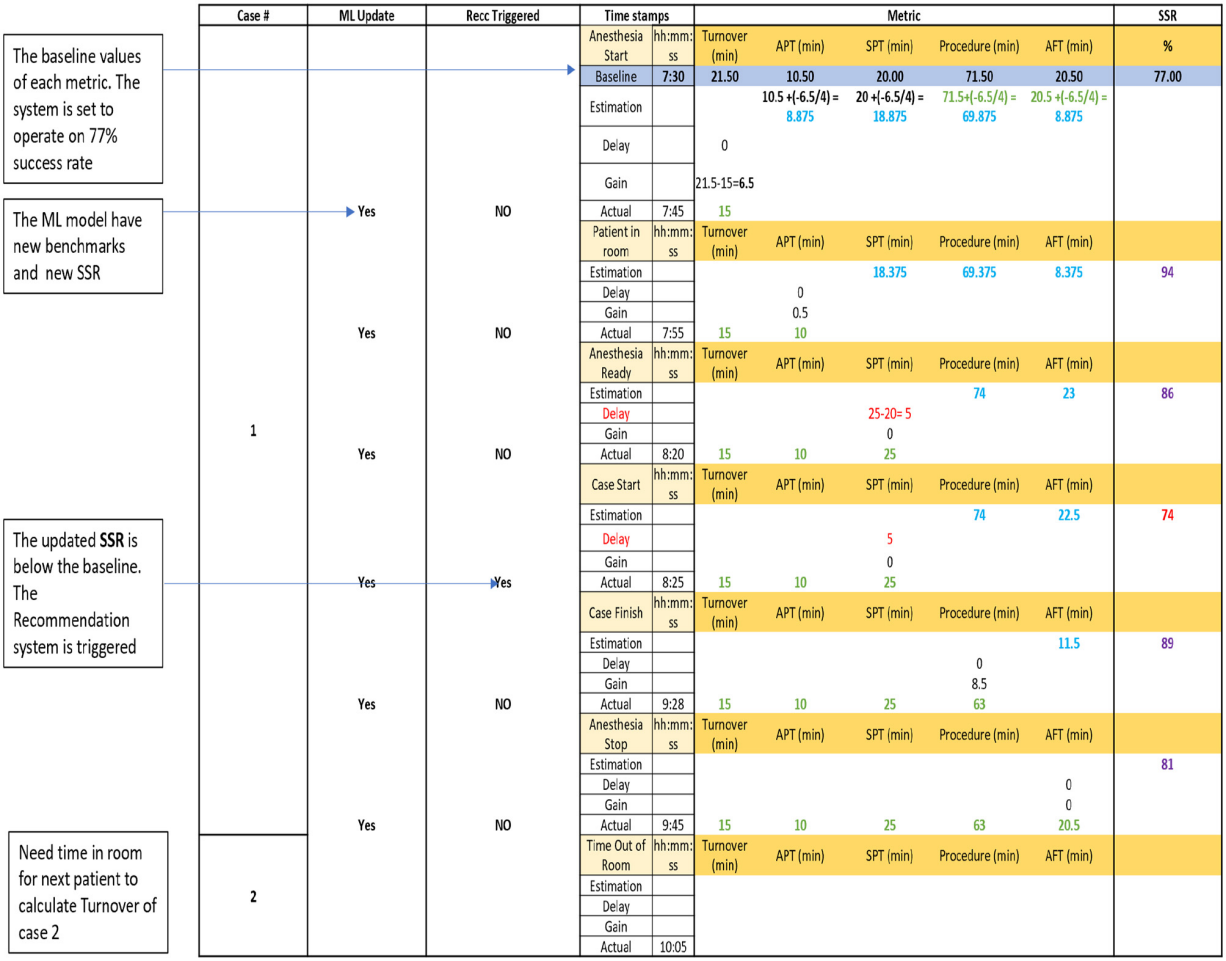
ML engine outputs updating the benchmarks and the SSR and recommendation system trigger time.

In Figure 2, the initial case for a surgical day is presented, highlighting instances of when and how the system updates the ML input, ML output, and the predicted SSR (Al Zoubi, 2024). The calculation formula is displayed in the first row to illustrate the application of the new metric formula. Additionally, it outlines specific points where the system activates the recommendation system to address potential delays. Metrics displayed in blue and green (actual values and estimated values) font serve as the ML model input and are updated with each new timestamp. The SSR is also updated whenever there is new ML input. The recommendation system is triggered only when the estimated SSR falls below the baseline value. This sequence repeats in the diagram until the completion of all four surgeries.

#### 3.3.2 The recommendation system component

The recommendation system specifically designed for the WM-FAST was created using a distinctive approach known as the positive deviance (PD) seminar described in detail in a previous publication (Gold et al., 2023). Introducing any other method for implementing a recommendation system could contribute to transforming the framework into a prescriptive analytics decision support system.

The decision to employ PD seminars as the source of recommendations for the WM-FAST, is in line with the objective of enhancing OR throughput by improving team efficiency. While alternative approaches, such as acquiring additional resources, hiring consultancy services, or adding personnel to oversee surgery flow, were available, they would have incurred extra costs. Moreover, the intention is to encourage the team's self-improvement by setting a positive example within the existing team dynamics and facilitating self-monitoring based on agreed-upon achievable and feasible performance standards.

The PD seminar, functioning as a recommendation system, underwent testing and validation independently of the ML engine. To illustrate, suggested recommendations were implemented in a 57-day trial focused on arthroplasty, and the outcomes are detailed in Table 4 (Al Zoubi, 2024).

**Table 4:** Walsh Test for the PD seminar recommendation implementation.

**Metric**	**APT**	**APT in room**	**SPT**	**CASE**	**SFT**	**AFT**	**Turnover**	**Success**
Mean-Before	0.028	0.011	0.008	0.044	0.003	0.004	29.625	0.140
Mean-After	0.026	0.009	0.009	0.042	0.004	0.005	26.785	0.640
STD-Before	0.009	0.006	0.004	0.006	0.001	0.002	7.856	0.350
STD-After	0.008	0.007	0.005	0.009	0.002	0.003	7.867	0.230
*t*-stat	1.386	1.835	−0.221	1.684	−1.475	−2.379	−0.387	−690.936
*p*-value	0.086	0.036	0.413	0.048	0.072	0.010	0.350	0.000
DF	46.000	58.000	63.000	72.000	71.000	61.000	74.000	37.000

### 3.4 WM-FAST: decision support system module

This module aims to enhance user experience and support workers in decision-making through the utilization of a dashboard and user interface. Initially, during the implementation of the WM-FAST, this aspect was manually executed. Benchmarks and SSR values were conveyed to practitioners without the aid of a dashboard. In cases of anticipated delays, surgeons had to resort to suggested actions from positive deviance seminars to manage subsequent stages. More information on the designed dashboard can be found in our previous work (Al Zoubi et al., 2023a).

### 3.5 WM-FAST translated in clinical practice

The goal for The Ottawa Hospital (TOH) was to achieve the highest possible SSR (i.e., completing 4 elective joint replacement surgeries, specifically hip and knee replacements) within a span of 8 h and without running into delays. The designated time frame for this endeavor was from 7:30 am to 3:30 pm. This target was set to avoid incurring overtime costs, which were estimated at $56.84 per minute, resulting in an annual expense of $600,000 per operating room/annually within the Division of Orthopedic Surgery at TOH alone. Despite various attempts and strategies, such as dedicating a room solely for hip and knee surgeries, implementing benchmarks for each stage of surgery, and exploring parallel processing by separating anesthesia activities, TOH has been unable to surpass a SSR of 39% since 2012.

The WM-FAST underwent implementation and validation at TOH's Riverside Campus, with the involvement of a team comprising seven arthroplasty surgeons, nurses, and anesthesiologists. Surgical operations were carried out on a weekly basis, utilizing two operating rooms every week, over approximately 23 Saturdays in 2023. The primary aim was to enhance operating room efficiency by optimizing overall team performance. Through the application of FAST, the clinical facility succeeded in increasing the success rate of arthroplasty surgeries from 39% to 93% over 23 weeks (Al Zoubi et al., 2024) (Table 5).

**Table 5:** Benchmarks and associated SSR (OR throughput).

**Scenario**	**APT**	**Case**	**AFT**	**Turnover**	**SSR**
1	< 10.5	53	< 20.5	< 21.5	93%
2	< 10.5	64	< 20.5	< 21.5	89%
3	< 10.5	< 71.5	< 20.5	< 21.5	59%
4	< 10.5	< 71.5	< 20.5	>21.5	69%
5	10.5–18.5	< 71.5	< 20.5	< 21.5	64%

## 4 Discussion

FAST is successfully able to improve the overall OR and complete more surgeries on time without changing clinical aspects, and without additional resources (human, financial, technical, etc.). This model leveraged data, artificial intelligence, stakeholder feedback and buy-in, and multiple ML models to not only predict, but suggest real time changes specific to the team and setting to achieve a better success rate. Quality of patient care and patient safety remained the top priority throughout the pursuit of this OR optimization solution that is a recommendation system which can be applied to other surgical procedures and other elements of healthcare delivery.

This work goes beyond the predictive analytics, to our knowledge, this work presents the first AI-driven framework to improve efficiency and productivity with:

1. The collaboration of key stakeholders: clinical team, data management team, hospital administration and patients.
2. Encompass all stages of the surgical process, including pre-operation, intra-operation, and post-operation phases.

FAST demonstrated its ability to be utilized in a multitude of settings, irrespective of the size of the team, and without the addition of resources. With the current status of surgery delays and scheduling issues, this is a potential model which can be implemented without the need for additional healthcare personnel, facilities or supplies. The optimization ability of the framework can allow for easier scheduling of complex cases across the week to improve the daily surgery success rate and minimize burnout in the healthcare team, which was a major issue during and post-covid (Murthy, 2022). For instance, in scenarios where two patients need scheduling across 5 days of surgery, with four patients each day, the framework can intelligently distribute cases based on factors such as procedure duration (e.g., prioritizing longer procedures for specific days), thus potentially enhancing weekly success rates. With the current delays in surgery, the need of the hour, is for an easily adoptable framework that can be integrated into the current system while providing setting specific real time suggestions.

One of the key factors in implementing change, especially in a healthcare setting in buy-in from the multidisciplinary team, with individuals truly being a core component of the influencing, being ready for and valuing the change (Nilsen et al., 2020). The success of the FAST framework is due in-large part to the integration of the clinical team in the process. The clinical team's commitment to the positive deviance seminars allowed them to take lead of this initiative and learn from the framework and each other by identifying key components and targets. Positive deviance focuses on why they succeeded, motivated the team and created a space for learning from team member's success. This was also beneficial for the implementation approach involving utilizing the framework for surgical team scheduling, with a focus on optimizing personnel combinations to predict and enhance surgical success rates.

## 5 Challenges and limitations

One of the most significant challenges in implementing this framework is that buy-in and support is required from three different healthcare disciplines. For example, if some nursing unions won't allow their nurses to begin work prior to 7:30 am in the morning, the surgery cannot begin on time as per the framework. Then there is the engineering layer connected to the development and deployment of these framework. Business owners have unique considerations and reservations compared to engineers and healthcare professionals, ranging from financial to human resource management. The conflicts of interest of these three healthcare stakeholders may slow down or impede the adoption and deployment of these framework in a healthcare institution.

A limitation inherent to this body of work is the inherent margin of error associated with uncertainty and ambiguity. Both epistemic and aleatoric uncertainty (dimensions) is worth considering. Epistemic uncertainty is common in ML models with limited, incomplete, or inappropriate training data. This uncertainty can be partially eliminated by improving the training data available. In contrast, aleatoric uncertainty is tied to measurement errors and randomness that can't be explained away. Some framework implementations such as surgical team scheduling framework can lead to team polarization. It can create two extremes, i.e., the strongest and the weakest members of the staff, which may lead to further division, and not allowing them to learn from each other. The patient scheduling application has drawbacks of its own, starting with the availability of the assigned surgeon for the day when the patient should optimally be scheduled (per the framework). Working around this constraint may significantly reduce the efficiency of the framework. For this application of the framework, the population was medically less complex, thus the surgeries were more likely to finish on time.

## 6 Conclusion

Prescriptive analytic frameworks, such as FAST are feasible and successful in utilizing real-time changes in variables and offering insights to improve OR efficiency and increase OR throughput. FAST's use of team and hospital specific data allow it to be adapted to a multitude of settings, including hospitals internationally and various surgical departments. A key component of the success of FAST was the integration of the multidisciplinary team as partners throughout the process.

## Data Availability

The datasets presented in this article are not readily available because this is an ongoing study. Requests to access the datasets should be directed to pfallavo@uottawa.ca.

## References

[B1] Al Zoubi F. (2024). Towards prescriptive analytics systems in healthcare delivery: AI-transformation to improve high volume operating rooms throughput (dissertation). University of Ottawa, Ottawa, ON, USA.

[B2] Al Zoubi F. (2024). Towards Prescriptive Analytics Systems in Healthcare Delivery: AI-Transformation to Improve High Volume Operating Rooms Throughput. Ottawa, ON, Canada: Université d'Ottawa/University of Ottawa.

[B3] Al Zoubi F. Beaulé P. E. Fallavollita P. (2023b). Factors influencing delays and overtime during surgery: a descriptive analytics for high volume arthroplasty procedures. Front. Surg. 10:1242287. 10.3389/fsurg.2023.1242287 38249310 PMC10797887

[B4] Al Zoubi F. Gold R. Poitras S. Kreviazuk C. Brillinger J. Fallavollita P.. (2023a). Artificial intelligence-driven prescriptive model to optimize team efficiency in a high-volume primary arthroplasty practice. Int. Orthop. 47, 343–350. 10.1007/s00264-022-05475-1 35759039

[B5] Al Zoubi F. Kashanian K. Beaule P. Fallavollita P. (2024). First deployment of artificial intelligence recommendations in orthopedic surgery. Front. Artif. Intell. 7:1342234. 10.3389/frai.2024.1342234 38362139 PMC10867959

[B6] Al Zoubi F. Khalaf G. Beaulé P. E. Fallavollita P. (2023c). Leveraging machine learning and prescriptive analytics to improve operating room throughput. Front. Digit. Health 5:1242214. 10.3389/fdgth.2023.1242214 37808917 PMC10556872

[B7] Al Zoubi F. Khalaf G. Beaulé P. E. Fallavollita P. (2023d). Leveraging machine learning and prescriptive analytics to improve operating room throughput. Front. Digit. Health 5:1242214. 10.3389/fdgth.2023.1242214 37808917 PMC10556872

[B8] American Society of Anesthesiologists (2020). Statement on ASA Physical Status Classification System. Schaumburg, IL: American Society of Anesthesiologists.

[B9] Bartek M. A. Saxena R. C. Solomon S. Fong C. T. Behara L. D. Venigandla R.. (2019). Improving operating room efficiency: machine learning approach to predict case-time duration. J. Am. Coll. Surgeons 229, 346–354.e3. 10.1016/j.jamcollsurg.2019.05.029 31310851 PMC7077507

[B10] Beaulé P. E. Frombach A. A. Ryu J-. J. (2015). Working toward benchmarks in orthopedic OR efficiency for joint replacement surgery in an academic centre. Can. J. Surg. 58:408. 10.1503/cjs.001215 26574833 PMC4651693

[B11] Belle V. Papantonis I. (2021). Principles and practice of explainable machine learning. Front. Big Data 4:688969. 10.3389/fdata.2021.688969 34278297 PMC8281957

[B12] Canadian Institute for Health Information (2024a). Wait times for priority procedures in Canada 2024. Available online at: https://www.cihi.ca/en/wait-times-for-priority-procedures-in-canada-2024 (Accessed August 15, 2025).

[B13] Canadian Institute for Health Information (2024b). Canadians waiting longer for priority surgeries and diagnostic imaging compared with pre-pandemic period. Available online at: https://www.cihi.ca/en/news/canadians-waiting-longer-for-priority-surgeries-and-diagnostic-imaging-compared-with-pre (Accessed August 15, 2025).

[B14] Cram P. Landon B. E. Matelski J. Ling V. Stukel T. A. Paterson J. M.. (2018). Utilization and short-term outcomes of primary total hip and knee arthroplasty in the United States and Canada. Arthritis Rheumatol. 70, 547–554. 10.1002/art.40407 29287312 PMC5876109

[B15] Fairley M. Scheinker D. Brandeau M. L. (2019). Improving the efficiency of the operating room environment with an optimization and machine learning model. Health Care Manage. Sci. 22, 756–767. 10.1007/s10729-018-9457-3 30387040

[B16] Gold R. Al Zoubi F. Brillinger J. Kreviazuk C. Garvin D. Schramm D.. (2023). Use of multidisciplinary positive deviance seminars to improve efficiency in a high-volume arthroplasty practice: a pilot study. Can. J. Surg. 66, E1–E7. 10.1503/cjs.018121 36596585 PMC9829057

[B17] Jain A. Patel H. Nagalapatti L. Gupta N. Mehta S. Guttula S.. (2020). “Overview and importance of data quality for machine learning tasks,” in Proceedings of the 26th ACM SIGKDD International Conference on Knowledge Discovery & Data Mining; Virtual Event (CA, USA: Association for Computing Machinery), 3561–3562. 10.1145/3394486.3406477

[B18] Maheshwari K. You J. Cummings K. C. 3rd Argalious, M. Sessler D. I. Kurz A.. (2017). Attempted development of a tool to predict anesthesia preparation time from patient-related and procedure-related characteristics. Anesth. Analg. 125, 580–592. 10.1213/ANE.0000000000002018 28430682

[B19] Murthy V. H. (2022). Confronting health worker burnout and well-being. New Engl. J. Med. 387, 577–579. 10.1056/NEJMp2207252 35830683

[B20] Nilsen P. Seing I. Ericsson C. Birken S. A. Schildmeijer K. (2020). Characteristics of successful changes in health care organizations: an interview study with physicians, registered nurses and assistant nurses. BMC Health Serv. Res. 20:147. 10.1186/s12913-020-4999-8 32106847 PMC7045403

[B21] Schiele J. Koperna T. Brunner J. O. (2021). Predicting intensive care unit bed occupancy for integrated operating room scheduling via neural networks. Naval Res. Logist. 68, 65–88. 10.1002/nav.21929

[B22] Sculley D. Holt G. Golovin D. Davydov E. Phillips T. Ebner D.. (2015). “Hidden technical debt in machine learning systems,” in Proceedings of the 28th International Conference on Neural Information Processing Systems, Vol. 2 (Montreal, QC, Canada: MIT Press), 2503–2511.

[B23] Waly F. J. Garbuz D. S. Greidanus N. V. Duncan C. P. Masri B. A. (2020). Safety of a ‘swing room' surgery model at a high-volume hip and knee arthroplasty centre. Bone Joint J. 102-B:112–115. 10.1302/0301-620X.102B7.BJJ-2019-1536.R1 32600199

